# Patient positioning on the operating table and patient safety: A systematic review and meta‐analysis

**DOI:** 10.1111/jan.16049

**Published:** 2024-01-07

**Authors:** Signe Berit Bentsen, Geir Egil Eide, Siri Wiig, Tone Rustøen, Cathrine Heen, Benedikte Bjøro

**Affiliations:** ^1^ Department of Health and Caring Sciences Western Norway University of Applied Sciences Bergen Norway; ^2^ SHARE Centre for Resilience in Healthcare, Faculty of Health Sciences University of Stavanger Stavanger Norway; ^3^ Division of Emergencies and Critical Care, Department of Research and Development Oslo University Hospital Oslo Norway; ^4^ Department of Nursing Science, Faculty of Medicine, Institute of Health and Society University of Oslo Oslo Norway; ^5^ Division of Emergencies and Critical Care, Department of Operating Services Oslo University Hospital Oslo Norway

**Keywords:** adverse events, harmful incidents, operating room, operating room nurse, operating room team, patient positioning, patient safety, systematic review; meta‐analysis

## Abstract

**Aim:**

To identify occurrence of harmful incidents related to patient positioning on operating table.

**Design:**

Systematic review and meta‐analysis.

**Data Sources:**

Eight databases including Ovid, Medline, Embase, CINAHL, the Cochrane Library, Epistemonikos, Scopus, Web of Science and Google Scholar were systematically searched from the inception of the databases to August 2023. Preferred Reporting Items for Systematic Reviews and Meta‐Analyses flow diagram depicting the flow information.

**Review Methods:**

The Cochrane Risk of Bias Tools were used to assess the risk of bias. Risk of harm with 95% confidence interval (CI) was estimated for each included study, and an overall risk was calculated using meta‐analysis.

**Results:**

Of the 22 included reports, two were randomized controlled trials (RCTs), five had a prospective cohort design, three had a cross‐sectional design, and 12 were register‐based studies. Intraoperative peripheral nerve injuries, perioperative pressure ulcers, musculoskeletal injuries, vascular injuries, postoperative pain and eye injuries were related to supine, lithotomy, Trendelenburg, prone and beach chair positioning. Overall risk of any harm was estimated as 0.2%. Studies with patients placed in prone positioning (8 study samples) had the highest risks of harm varying from 0.19 to 0.81, with an overall risk of 0.33. Meta‐analysis of the two RCTs showed higher risk of chemosis with head‐down positioning than with head in neutral position (overall relative risk = 1.64; 95% CI: [1.25, 2.14]).

**Conclusions:**

Harmful incidents related to patient positioning occur and consequences can be severe. The operating room teams should be aware of the harms and prevent and treat them seriously.

**Impact:**

This review underlines that research is sparse on patient positioning on operating table and harmful incidents. There is a need for high‐quality, well‐designed studies that focus on harmful incidents and prevention of harm related to patient positioning.

**Patient or Public Contribution:**

No patient or public contribution, as this is a review of previous research.

## 1 INTRODUCTION

Patient safety involves reducing the risk of unnecessary harm associated with healthcare to an acceptable minimum. A harmful incident, or an adverse event, is an injury related to medical management, in contrast to complications of a disease, and may be preventable or not. Medical management includes all aspects of care, including diagnosis and treatment, failure to diagnose or treat, and the systems and equipment used to deliver care (World Health Organization [WHO], 2005).

The operating room is known to be one of hospitals' high‐risk environments and unsafe medical care appears (Bates et al., 2023; Jha et al., 2010; WHO, 2019). Proper patient positioning is a critical part of any operation and is how the patient is placed on the operating table. Optimal patient positioning is important to provide access to the surgical field and may minimize the risk of harm (Fawcett, 2019). Positioning the patient for surgery involves teamwork (i.e. between the surgeon, anaesthesiologist, anaesthesia nurse and operating room nurse), where the operating room nurse plays a key role in protecting the patient from harm (Bjøro et al., 2022; Brooker et al., 2020). Supine, prone and lateral positioning are standardized procedures for ensuring appropriate safe patient positioning for operating room teams. Since surgical procedures have become increasingly technically complex (e.g. robotic‐assisted laparoscopic surgery) more challenging patient positions are required. Therefore, modifications are needed to allow for different procedures and individual needs (Fawcett, 2019). Harmful incidents occur in these settings, even though they are often preventable. The pressure, shear and friction forces used to establish and maintain a position can harm the skin and underlying tissue (Das et al., 2019; Gefen et al., 2020). Eye injuries are rare but do occur. Direct pressure on the eyes might increase intraocular pressure leading to ischemic optic neuropathy. Direct pressure on the eye combined with hypotension may cause retinal artery occlusion with temporary or permanent blindless (Fawcett, 2019). The musculoskeletal system may be exposed to stress as anaesthetics and muscle relaxants depress pain, pressure receptors and muscle tone can lead to intraoperative peripheral nerve injury (Laughlin et al., 2020).

Incidence of intraoperative peripheral nerve injury with Trendelenburg position ranges between 0.16% and 11% (Bjøro et al., 2020; Cornelius et al., 2021; Oblak & Gillespie, 2021). In addition, occurrence of intraoperative pressure ulcer varies from 12% to 66% due to different patient positions (Gefen et al., 2020). Eye injury after non‐ocular surgery is rare but is estimated to be between 0.0002% and 0.05% (Maerz et al., 2017; Ripa et al., 2022). Furthermore, occurrence of musculoskeletal pain due to non‐surgical procedures ranges from 1.5% to 89% (Gefen et al., 2020). Preexisting risk factors are associated with positioning injuries. Comorbidities such as vascular disease and diabetes mellitus, higher American Society of Anesthesiologists score, body mass index and skin‐to‐skin time (from incision to closure) are associated with a higher occurrence of intraoperative peripheral nerve injury in those placed in Trendelenburg position (Bjøro et al., 2020; Cornelius et al., 2021; Oblak & Gillespie, 2021). Blood loss, higher core temperature and previous skin problems are associated with intraoperative pressure ulcer in those in prone position (Choi et al., 2022; Techanivate et al., 2020).

A preliminary search of PROSPERO, Epistemonikos, Medline and the Cochrane Library Database of Systematic Reviews was conducted, and no current systematic review of patient positioning on the operating table and occurrence of harmful incidents was in progress or identified. To our knowledge, this is the first systematic review with meta‐analysis identifying occurrence of harmful incidents related to different patient positions on operating table. The operating room is a high‐risk environment and harmful incidents appear. Thus, it is important to identify harmful incidents related to patient positioning on operating table, which is the rationale behind the current study.

## 2 AIM

To identify occurrence of harmful incidents related to patient positioning on operating table.

## 3 METHODS

### 3.1 Design

This systematic review was conducted based on the Cochrane Handbook for Systematic Reviews of Interventions (Higgins et al., 2019). We report in accordance with the Preferred Reporting Items for Systematic Reviews and Meta‐Analyses (PRISMA 2020 statement) (Page et al., 2021) (Appendix S1).

### 3.2 Eligibility criteria

Eligible study designs included published, peer‐reviewed quantitative studies in English, Norwegian, Danish or Swedish. No limits were set for the year of publication. Participants were patients (≥18 years) undergoing surgery in supine, prone or lateral positions with or without modifications. Studies were excluded if participants were cadavers or volunteers, surgical examination, radiological interventions or endoscopies. The outcomes were occurrence of harmful incidents related to patient positioning on operating table (i.e. intraoperative peripheral nerve injury, musculoskeletal injury, vascular injury, skin injury and eye injury).

### 3.3 Data searches

The search was performed in the following databases: Ovid, Medline, Embase, CINAHL, the Cochrane Library, Epistemonikos, Scopus, Web of Science and Google Scholar. The search was performed February 22, 2022, and rerun December 13, 2022, and August 18, 2023. Manual screening of reference lists and a forward citation search in Google Scholar of the included reports was performed. The PICO (Population, Interventions, Comparisons, Outcomes) framework was employed to develop the search strategy (Higgins et al., 2019). Search terms were related to population, intervention and outcomes, and included patients undergoing surgery, patient positioning on operating table and harmful incidents. The full search strategy for all databases is listed in Appendix S2.

### 3.4 Selection

**FIGURE 1:**
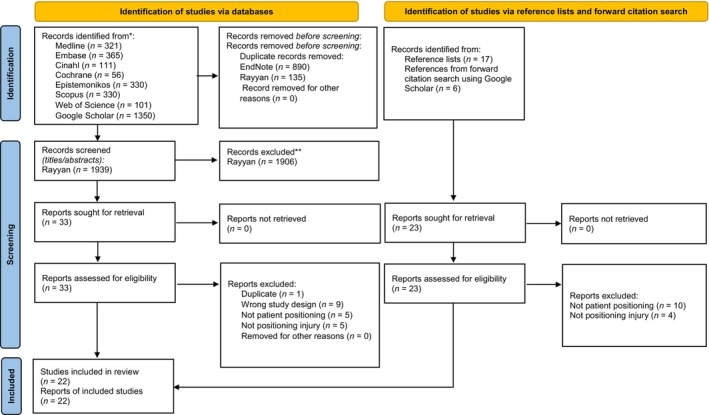
Flow diagram for the systematic review. *Consider, if feasible to do so, reporting the number of records identified from each database or register searched (rather than the total number across all databases/registers). **If automation tools were used, indicate how many records were excluded by a human and how many were excluded by automation tools. From: Page et al. (2021).

All records were imported into EndNote library and web application Rayyan (https://rayyan.qcri.org/) for storage, organization, blinding and screening of titles and abstracts for the designated systematic review. A total of 2964 records were obtained by searching the electronic databases and 1939 records remained after excluding duplicate articles. Based on the eligibility criteria for the review, 1906 articles were excluded by screening titles and abstracts, and 33 articles were selected for full‐text assessment. The screening process is summarized in the PRISMA flow diagram (Page et al., 2021) (Figure 1). Two researchers (SBB, BB) independently screened titles, abstracts and full‐text articles for inclusion. Any disagreement was resolved by consensus.

### 3.5 Data extraction

Data extraction was performed by two researchers (SBB, BB) in Google Forms (Higgins et al., 2019). The following data items were extracted: (1) Publication characteristics: authors, year of publication, country, aims and ethical approval; (2) Methodology: design, sample size, characteristics of the populations and data collection; and (3) Key findings related to the review question: patient positioning on operating table (i.e. supine, prone, lateral) and harmful incidents (i.e. intraoperative peripheral nerve injury, musculoskeletal injury, vascular injury, skin injury, eye injury).

### 3.6 Quality assessment

The Cochrane tools for assessing risk of bias (RoB‐2, ROBINS‐1) (Higgins et al., 2019) were utilized to assess the methodological quality of included studies. RoB‐2 covers bias in randomization process, deviations from intended interventions, missing outcome data, measurement of the outcome and selection of reported results (Higgins et al., 2019; Sterne et al., 2019), while ROBINS‐1 covers bias in confounding, selection of participants, classification of intervention, deviations from intended interventions, missing data, measurement of the outcome and selection of the reported result (Higgins et al., 2019; Sterne et al., 2016). Two researchers (SBB, BB) independently evaluated risk of bias. Any disagreement was resolved by consensus.

### 3.7 Synthesis

Meta‐analyses were performed in Stata 17 using the metan command. For each included study the risk of harm was estimated with 95% confidence interval (CI), illustrated with forest plots, and the pooled effect and heterogeneity index *I* ^2^ (Higgins et al., 2019) was calculated. The included studies had not the same true effect size as they used different designs, sample sizes, outcome measures and different patient positions on operating table. Therefore, the random‐effects model was considered the most correct choice. The results of the meta‐analysis are reported using the risks and relative risks (RRs) and 95% CIs.

The results are grouped in (1) harm related to supine positioning (i.e. supine, lithotomy, Trendelenburg, beach chair); (2) harm related to prone positioning; and (3) harm related to lateral positioning (Fawcett, 2019).

## 4 RESULTS

### 4.1 Description of the reports

A total of 22 papers were included. Of these, 12 (55%) were register‐based studies. Two were randomized controlled trials (RCTs), five had a prospective cohort design, and three had a cross‐sectional design. The 22 papers included supine, lithotomy, Trendelenburg, beach chair, prone and lateral positioning (Table 1).

**TABLE 1:** Study characteristics, aims, methodology, operation, patient position and ethical approval (*n* = 22).

Author, year, country of origin	Aims	Study design (SD), sample size (Sz)	Outcome measurement	Operation	Patient positioning	Ethical approval
Jeon et al. (2007); Korea	Evaluate the effect of head position on postoperative chemosis after prone spinal surgery and determine which factors that contribute to the development of postoperative chemosis	SD: Randomized controlled trial (RCT). Sz: Total: *n* = 108 HN: *n* = 54 HD: *n* = 54	*Chemosis*: yes/no *Degree of chemosis*: A 4‐point Likert scale: None Mild: nasal or temporal Moderate: nasal and temporal Severe: total, corneal involvement *Procedure*: Scored by an anaesthetist postoperative	*Lumbar spine surgery*: Interbody fusion: *n* = 22 Laminectomy: *n* = 46 Discectomy: *n* = 40	*Prone positioning*: Positioned prone on a Wilson frame Head was placed on a prone headrest without external direct compression to both eyes The neck was in midline position HN: The imaginary line from the occipital protuberance to the top of C7 spine process is parallel to the operating table. A hardboard with height of 5 cm beneath the prone headrest and by adding some towels if necessary HD: Similar to HN, but 5 cm lower than HN	Institutional review board
Mohammadi and Hosseini (2008); Iran	Evaluate factors that contribute to the development of chemosis after spine surgery in prone position	SD: RCT Sz: Total: *n* = 140 HN: *n* = 70 HD: *n* = 70	*Chemosis*: yes/no *Degree of chemosis*: A 4‐point Likert scale: None Mild: nasal or temporal Moderate: nasal and temporal Severe: total, corneal involvement *Procedure*: Scored by an anaesthetist postoperative	*Lumbar spine surgery*: Prone spinal surgery	*Prone positioning*: Positioned prone on a Wilson frame Head was placed on a prone headrest without external direct compression to both eyes The neck was in midline position HN: The imaginary line from the occipital protuberance to the top of C7 spine process is parallel to the operating table. A hardboard with height of 5 cm beneath the prone headrest and by adding some towels if necessary HD: Similar to HN, but 5 cm lower than HN	Institutional review board
Lin et al. (2017); Singapore	Analyse the prevalence and predictors of pressure injuries from spine surgeries performed in the prone position	SD: Register‐based study^a^ Sz: *n* = 209	*Per‐operative pressure ulcer*: National Pressure Ulcer Advisory Panel staging clinical practice guidelines *Procedure*: The data were independently collected by two orthopaedic surgeons	*Spine surgery*: Anterior approach: *n* = 26 Posterior approach: *n* = 183 Spinal deformity: *n* = 58 Lumbar PID: *n* = 53 Cervical myelopathy: *n* = 16 Lumbar spinal stenosis: *n* = 50 Spondylolisthesis: *n* = 27 Spinal metastasis: *n* = 6	*Prone positioning*: Jackson table The head was placed on a padded headrest and the chest on a bolster. The anterior superior iliac spines and thighs on cushioned side support. Knees were flexed and rested on gel pad. Both shoulder and elbows were flexed to 90° and rested on foam‐padded arm boards	Local ethics board committee
Techanivate et al. (2020); Thailand	Identify the incidence and independent risk factors associated with the occurrence of facial pressure ulcer in patients who underwent spine surgery in prone position using padded devices to support the face that least ≥3 h	SD: Register‐based study^a^ Sz: *n* = 300	*Facial pressure ulcer*: National pressure ulcer advisory panel staging clinical practice guidelines *Procedure*: The data were collected by the orthopaedic surgeons at the institute	*Spine surgery*: Laminectomy and fusion: *n* = 248 Minimal invasive surgery: *n* = 52 Degenerative spine: *n* = 212 Tumour/infection: *n* = 55 Scoliosis: *n* = 33	*Prone positioning*: Before turning into the prone position patients’ face, chest, iliac crest and knees were moisturized with 3 mol/L Cavilon Durable Barrier cream and then the head padded device was placed on their face After prone eyes were checked up to rule out globe compression Two types of facial support devices were used (UM Prone headrest and ProneView)	The ethics committee
Choi et al. (2022); Korea	Investigate the incidence of perioperative pressure injuries and identify factors associated with hospital‐acquired pressure injury in patients having general anaesthesia for spine surgery in prone positioning	SD: Cohort study. Sz: Total: *n* = 287	*Per‐operative pressure ulcer*: National pressure ulcer advisory panel staging clinical practice guidelines *Procedure*: The presence or absence of PPU was carefully assessed by the attending anaesthesiologist and spine surgeon who participated only in this assessment of PPU and not in other parts of the study	*Spine surgery*: Discectomy: *n* = 18(6%) Decompression: *n* = 193(67%) Decompression with instrumentation: *n* = 76(27%)	*Prone positioning*: Chest pad: *n* = 79(28%) Jackson spine table: *n* = 171(60%) Wilson spine table: *n* = 37(13%)	Institutional review board
Nilsson (2013); Sweden	Explore the association of supine position to postoperative positioning pain and pressure ulcers in adult patients receiving general anaesthesia	SD: Cross‐sectional study Sz: *n* = 86	*Postoperative pain*: *Pain occurrence* (yes/no): Heels, arms, overall related to the operation *Pain intensity*: *NRS*: 0 = no pain to 10 = severe pain *Per‐operative pressure ulcer*: *Grade I*: Non‐blanchable erythema, with intact skin surface *Grade II*: Epithelial damage, abrasion, or blister *Grade III*: Abrasion or blister *Grade IV*: Full thickness of skin with deep cavity *Procedure*: Collected in the PACU by a nurse	*Ear, nose, throat surgery*: *n* = 27 *Plastic surgery*: *n* = 27 *Eye surgery*: *n* = 20 *Oral surgery*: *n* = 12	*Supine positioning*: Not described	Department of anaesthesia at the hospital
Warner et al. (1994); USA	Determine the frequency of lower extremity neuropathies with persistent motor deficit after procedures performed on patients in a lithotomy position	SD: Register‐based study^a^ Sz: *n* = 220.	*Neuropathy determined by one of five major lower‐extremity nerves*: Peroneal nerve Tibial nerve Sciatic nerve (combined common peroneal and tibial) Femoral nerve Obturator nerve *Procedure*: Not reported	All surgical operations in lithotomy positioning	*Lithotomy positioning*: Not described	Institutional review board
Warner et al. (2000); USA	Determine the frequency and natural history of symptoms of lower extremity neuropathies after surgery in lithotomy position	SD: Cohort study Sz: *n* = 991	*Neuropathy*: *Procedure*: Standardized questionnaire: lower extremity neuropathy Standardized neurologic examination	*Urological surgery*: *n* = 377/991 (38%) *Gynaecological surgery*: *n* = 317/991 (32%) *Gastroenterological surgery*: *n* = 188/991 (19%) *Other*: *n* = 109/991 (11%)	*Lithotomy positioning*: Lithotomy: low, standard, high Degree: not reported Stirrups: candy cane, knee crutch, boot support	Institutional review board
Ulm et al. (2014); USA	Evaluate the incidence and risk factors of positioning‐related injury in gynecologic robotic surgery	SD: Register‐based study^a^ Sz: *n* = 831	*Positioning‐related injuries*: Injuries that were not present prior to surgery and presented within the 2 first weeks after surgery *Neuropathy*: Symptoms of paraesthesia, dysesthesia, or weakness in the distribution of upper or lower extremity nerve *Vasculopathies*: Hematomas, ecchymoses, or compartment syndrome in upper or lower extremity *Musculoskeletal*: Ligamentous, tendentious, bony, or muscular injury *Procedure*: Patient charts were reviewed	*Gynecologic robotic‐assisted surgery*: Pelvic and periaortic lymph node dissection Salpingo‐oophorectomy. Hysterectomy Pelvic lymph node dissection. Radical trachelectomy	*Trendelenburg positioning*: Trendelenburg degree: not reported Lithotomy: the leg in stirrups, the knee flexed to an angle of approximately 90° The arms were held parallel to the body	Institutional review board
Gezginci et al. (2015); Turkey	Evaluate postoperative pain and neuromuscular complications (i.e. hip abduction, flexion weakness, pain or sensitivity on upper and lower extremities), which are related to prolonged exposure to steep Trendelenburg and low‐lithotomy position during RALP	SD: Register‐based study^a^ Sz: *n* = 534	*Neuromuscular injury*: *Symptoms*: Occurrence Locations *Postoperative pain*: Occurrence Locations Intensity (VAS) *Procedure*: Not reported	*Urologic robotic‐assisted surgery*: RALP	*Trendelenburg positioning*: Trendelenburg: 15–20° Lithotomy: legs separated in flexion and abduction, level not reported The arms were held parallel to the body Shoulder support used to prevent sliding downward	The cancer centre approval
Johansson and von Vogelsang (2019); Sweden	Describe patient‐reported extremity symptoms after RALC in patients with bladder cancer	SD: Cohort study Sz: *n* = 94	*Postoperative pain and unspecific symptoms*. *Upper extremity*: Symptoms, function and disabilities in the arm, shoulder and hand (QuickDASH) *Lower extremity*: Functional status in the presence of the lower extremity musculoskeletal problems (LEFS) *Per‐operative pressure ulcer*: National pressure ulcer advisory panel staging clinical practice guidelines *Procedure*: Standardized and study‐specific questionnaires Perioperative nurses assess the patient closely for signs of intraoperative skin and musculoskeletal injury	*Urologic robotic‐assisted surgery*: Robotic‐assisted cystectomy in patients with bladder cancer	*Trendelenburg positioning*: Trendelenburg: degree 35° Lithotomy: legs separated and secured with straps Both arms cradled in beanbag	The regional board for ethics of research
Wen et al. (2014); USA	Characterize the complications related to steep Trendelenburg positioning and lithotomy positioning nationally	SD: Register‐based study^a^ Sz: *n* = 175,699	*ICD‐9‐CM codes*: Nerve injury Compartment syndrome Eye injury *Procedure*: Not reported	*Urological surgery*: RARP: *n* = 61,656 (35%) LRP: *n* = 2682 (2%) ORP: *n* = 111,361 (63%)	*Trendelenburg positioning*: Trendelenburg: degree not reported Lithotomy: level not reported	Not reported
Mills et al. (2013); USA	Determine incidence of positioning injury during robotic‐assisted urological surgery, identify risk factors and describe the time to resolution of the neurological injury	SD: Register‐based study^a^ Sz: *n* = 334	*Neuropathy*: Incidence of upper or lower extremity neuropathy *Time to resolution of the injury* *Procedure*: Each patient chart was carefully reviewed for documentation of any new complaint from the PACU	*Urologic robotic‐assisted surgery*: Prostatectomy: *n* = 137 Partial nephrectomy: *n* = 58 Pyeloplasty *n* = 24 Adrenalectomy: *n* = 6	*Trendelenburg positioning* Trendelenburg: degree: 30° Lithotomy in stirrups Level lithotomy: not reported The arms were held parallel to the body Beanbag used in 33% of cases	Institutional review board
Mattei et al. (2013); Switzerland	Evaluate whether positioning injuries as indicated by serum‐CK levels occurs in patients undergoing RARP and ePlnd with prolonged steep Trendelenburg position	SD: Cohort study. Sz: *n* = 60	*Per‐operative pressure ulcer*: *Stage I*: Finger pressure causes red skin to disappear; spontaneous healing within 3 days *Stage II*: Finger pressure does not cause red skin to disappear; healing within 10 days under local therapies *Stage III*: Evident skin lesion; healing within 3 months under local and systematic therapies *Neuromuscular injury* *Postoperative pain* *Procedure*: Patients were examined daily *Rhabdomyolysis*: Serum‐CK level on postoperative Day 1 after RARP >5000 IU/L	*Urologic robotic‐assisted surgery*: RARP ePlnd	*Trendelenburg positioning*: Trendelenburg: degree: 30° Lithotomy: divaricated 30°	Not reported
Tsubouchi et al. (2022); Japan	Clarify the relationships of the intraoperative surgical position with the occurrence rate of postoperative rhabdomyolysis and with postoperative renal function	SD: Cross‐sectional study Sz: Total: *n* = 276 Trendelenburg with opened legs: *n* = 130 Trendelenburg with lithotomy: *n* = 146	*Rhabdomyolysis*: Serum‐CK level on postoperative Day 1 after RARP >1000 IU/L *Compartment syndrome*: Subjective symptoms of pain, paraesthesia and cold feeling of skeletal muscle	*Urologic robotic‐assisted surgery*: RARP	*Trendelenburg positioning*: *Trendelenburg positioning with opened legs*: Trendelenburg: degree 23°–25° Opened legs: the legs were placed on a split‐leg table *Trendelenburg positioning with lithotomy*: Steep Trendelenburg: degree 23°–25° Lithotomy: the legs flexed 90° at the hips. The knees are bent at 70°–90°	Institutional ethics committee
Levy et al. (2019); USA	Determine the prevalence and risk factors of LFCN in obese patients following shoulder surgery in the beach chair position	SD: Register‐based study^a^ Sz: *n* = 400	*Neurapraxia*: Numbness Tingling Pain located in the anterolateral thigh Clinical muscle function of intact and symmetric quadriceps *Procedure*: Clinical examination	*Shoulder surgery*: Shoulder arthroplasty for any aetiology: *n* = 113 All other open surgery (i.e. fracture fixation): *n* = 23 Arthroscopic rotator cuff repair: *n* = 214 All other arthroscopic shoulder surgery: *n* = 50	*Beach chair positioning*: *Position 1*: Upright position Flexion at the hips of 60° to 70° Knees flexed 30° Heels were padded Well‐padded kidney rests Belt across the thighs *Position II*: Hip flexed 45° Use of reverse Trendelenburg Thick foam padding between abdomen and thigh A belt was placed perpendicular to the thigh	Review board approval
Grant et al. (2019); USA	Describe the intraoperative peripheral nerve injury in the context of surgical service, patient position, injured nerve, severity of injury and indemnity amount	SD: Register‐based study^a^ Sz: *n* = 75	*Intraoperative peripheral nerve injury*: *NAIC*: Temporary (NAIC): 0–4 Permanent (NAIC): 5–9 *Procedure*: Not reported	*Orthopaedic surgery* *Gynecologic surgery* *General surgery* *Cardiac surgery* *Other surgeries*	*Different positions*: Lateral positioning Lithotomy positioning Prone positioning Supine positioning Other	Not reported
Bohrer et al. (2009); USA	Determine the overall incidence of pelvic nerve injury following both major and minor benign and oncologic gynecologic surgical procedure at a single academic medical centre	SD: Cohort study Sz: *n* = 616	*Intraoperative peripheral nerve injury*: *Motor strength*: Hip, knee and ankle strength MRC rating scale: 0 (absence of any resistance) to 5 (being full strength) *Sensation*: Abdomen, lower and upper extremities Light touch and pinprick *Reflexes*: Patellar and ankle jerk reflexes *Questionnaires*: Abdomen, pelvis, lower extremities: Pain, loss of sensation and/or abnormal sensation (e.g. paraesthesia) Hip, knee, ankle joint: Feeling weakness	*Gynaecological surgery*: Laparotomy (22%) Vaginally (43%) Laparoscopy (26%) Other (19%)	*Supine positioning* (6%) *Lithotomy positioning* (94%): Level: low or high, Stirrups: boots (46%) Level: high, Stirrups: candy cane (47%)	Institution review board
Navarro‐Vicente et al. (2011); USA	Determine the incidence of IPNI after abdominal colorectal open and laparoscopic surgery	SD: Cross‐sectional study Sz: Total: *n* = 2304 Supine positioning: *n* = 2211 Trendelenburg positioning: *n* = 93	*Intraoperative peripheral nerve injury*: Clinical peripheral neurological deficit proven by EMG	*Open or laparoscopic gastroenterological surgery*: Sigmoidectomy Total colectomy Subtotal colectomy Ileocecal resection	*Supine positioning (1996–2006)*: Upper limb abducted at an angle below 90° *Trendelenburg positioning (2007–2009)*: Trendelenburg: degree not reported Lithotomy in stirrups Level lithotomy: Not reported Both arms tucked to their side Shoulder restrains	Institution review board
Dinaux et al. (2017); USA	Investigate the effect of patient positioning during abdominal perineal resection on perineal wound infections and dehiscence	SD: Register‐based study^a^ Sz: *n* = 149	*Perineal wound infection*: Infection reported in discharge reports or follow up notes within 3 months *Postoperative wound dehiscence*: Separated wound edges without obvious sign of infection. Reported in discharge report or follow up within 3 months *Procedure*: Not reported	*Gastroenterological surgery*: Abdominal perineal resection: Miles operation	*Lithotomy positioning (n = 91)*: Not described *Prone jack‐knife positioning (n = 58)*: Not described	Not reported
Ueno et al. (2020); Japan	Investigate the frequency of ulcers caused by pelvic positioner in hip surgeries and identify the risk factors specifically associated with the use of a pelvic positioner	SD: Register‐based study^a^ Sz: *n* = 229	*Postoperative pressure ulcer*: Classified according to the National pressure ulcer advisory panel staging clinical practice guidelines *Procedure*: Two nurses and one doctor assessed the patients’ skin through the perioperative period	*Orthopaedic surgery*: Total hip arthroplasty: Patients: *n* = 229 Hip surgeries: *n* = 265^b^	*Lateral positioning*: Upper extremities: placed on armrests with gel pads under axilla, secured with urethane Nonoperative lower extremity: secured with urethane foams and bands Pelvic positioner: support over the sacrum, support over the pubic symphysis, double support over iliac spine	Not reported
Yoshimura et al. (2016); Japan	Clarify the risk factors for intraoperative acquired pressure ulcers in the park bench position	SD: Register‐based study^a^ Sz: *n* = 277	*Perioperative pressure ulcer*: No signs of ulcer at the time of admission and the appearance of redness immediately after surgery that remained 24 h later	*Cerebellum surgeries*: Cerebellopontine angle tumour: *n* = 266 Microvascular decompression: *n* = 11	*Park bench positioning*: Patient placed in lateral positioning Head is clamped in a May‐field frame Lateral trunk is fixed on the main operating table Upside arm is positioned along the lateral trunk Downside arm fixed on an external arm board	Institutional review board

Abbreviations: CK, creatinine kinase; EMG, electromyography; ePlnd, extended pelvic lymph node dissection; FPU, facial pressure ulcer; h, hour; HD, head down; HN, head neutral; IPNI, intraoperative peripheral nerve injury; LFCN, lateral femoral cutaneous nerve palsy; LEFS, lower extremity functional scale; LRP, laparoscopic radical prostatectomy; MRC, Medical Research Council; NAIC, national association of insurance commissioners; NRS, numeric rating scale; RALC, robot‐assisted laparoscopic cystectomy; ORP, open radical prostatectomy; PACU, postoperative anaesthesia care unit; PID, prolapsed intervertebral disc; PPU, per‐operative pressure ulcer; Quick DASH, disability in the arm, shoulder and hand; RALP, robot‐assisted laparoscopic radical prostatectomy; RARP, robotic‐assisted radical prostatectomy; RAS, robotic‐assisted laparoscopic surgery; SD, side docking; TD, traditional docking; VAS, visual analogue scale.

^a^ Retrospective view of prospective data.

^b^ Some have more than one hip surgery.

### 4.2 Risk of bias in included papers

**FIGURE 2:**
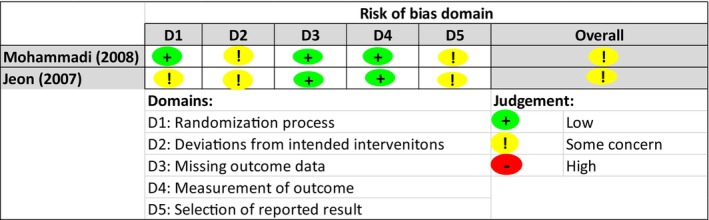
Within studies risk of bias assessment‐2 for RCTs on five criteria and overall domains.

**FIGURE 3:**
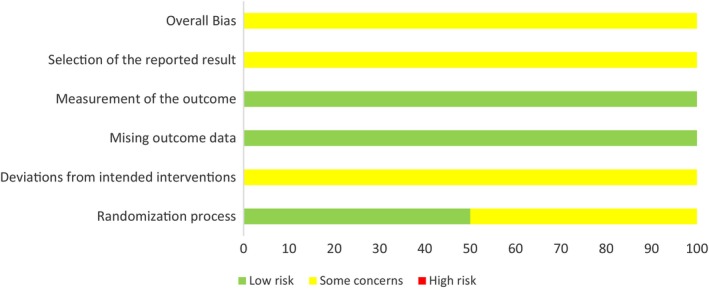
Risk of bias assessment‐2 graph summary plot: review authors' judgements about each RoB‐2 item and overall bias presented as percentages across the two RCTs.

Among the included papers, the two RCTs were appraised using RoB‐2 (Jeon et al., 2007; Mohammadi & Hosseini, 2008). Detailed descriptions of the RoB‐2 domains are presented as RoB summary and RoB graph (Figures 2 and 3). Twenty non‐randomized studies (Bohrer et al., 2009; Choi et al., 2022; Dinaux et al., 2017; Gezginci et al., 2015; Grant et al., 2019; Johansson & von Vogelsang, 2019; Levy et al., 2019; Lin et al., 2017; Mattei et al., 2013; Mills et al., 2013; Navarro‐Vicente et al., 2011; Nilsson, 2013; Techanivate et al., 2020; Tsubouchi et al., 2022; Ueno et al., 2020; Ulm et al., 2014; Warner et al., 1994, 2000; Wen et al., 2014; Yoshimura et al., 2016) were assessed with ROBINS‐1 (Table 2).

**TABLE 2:** Within risk of bias assessment (ROBINS‐1) for non‐randomized studies on summary of risk of bias on seven criteria and overall.

Study	Bias due to confounding	Bias in selection of participants into the study	Bias in classification of interventions	Bias due to deviations from intended interventions	Bias due to missing data	Bias in measurement of outcomes	Bias in selection of the reported result	Overall bias
Warner et al. (1994)	Moderate	Serious	Serious	NI	Low	Moderate	Moderate	Serious
Warner et al. (2000)	Moderate	Low	Low	Moderate	Low	Low	Moderate	Moderate
Bohrer et al. (2009)	Moderate	Low	Serious	Moderate	Moderate	Moderate	Moderate	Serious
Navarro‐Vicente et al. (2011)	Serious	Serious	Serious	Serious	Moderate	Moderate	Moderate	Serious
Mattei et al. (2013)	Serious	NI	Low	Moderate	Low	Low	Moderate	Serious
Mills et al. (2013)	Moderate	Moderate	Moderate	Moderate	Low	Moderate	Moderate	Moderate
Nilsson (2013)	Serious	Low	Serious	Serious	Low	Moderate	Moderate	Serious
Wen et al. (2014)	Serious	Serious	Moderate	NI	Low	Moderate	Moderate	Serious
Ulm et al. (2014)	Moderate	Moderate	Moderate	Moderate	Low	Moderate	Moderate	Moderate
Yoshimura et al. (2016)	Moderate	Low	Low	Moderate	Low	Moderate	Moderate	Moderate
Gezginci et al. (2015)	Moderate	Moderate	Moderate	Moderate	Low	Moderate	Moderate	Moderate
Lin et al. (2017)	Moderate	Moderate	Moderate	Moderate	Low	Moderate	Moderate	Moderate
Dinaux et al. (2017)	Moderate	Moderate	Moderate	Moderate	Low	Moderate	Moderate	Moderate
Levy et al. (2019)	Moderate	Serious	Moderate	Moderate	Low	Moderate	Moderate	Serious
Johansson and von Vogelsang (2019)	Moderate	Moderate	Low	Moderate	Low	Moderate	Moderate	Moderate
Grant et al. (2019)	Critical	Critical	NI	Serious	Low	Moderate	Moderate	Critical
Ueno et al. (2020)	Moderate	Moderate	Moderate	Moderate	Low	Moderate	Moderate	Moderate
Techanivate et al. (2020)	Moderate	Moderate	Moderate	Moderate	Low	Moderate	Moderate	Moderate
Tsubouchi et al. (2022)	Moderate	Moderate	Low	Moderate	Low	Low	Moderate	Moderate
Choi et al. (2022)	Moderate	Moderate	Moderate	Moderate	Serious	Moderate	Moderate	Serious

### 4.3 Meta‐analysis results

**FIGURE 4:**
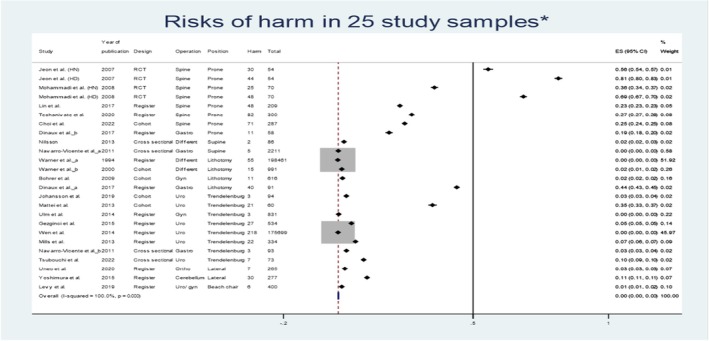
Forest plot of 25 samples in 22 outcome reports until August 2023. *Study samples >22 because multiple outcome measurements are used in some reports. Forest plot of the risk of harm in 25 samples from 22 reports until August 2023. Number of samples (25) are higher than the number of reports (22) because two reports (Jeon et al., 2007; Mohammadi & Hosseini, 2008) were RCTs each with two independent groups and two reports evaluated two different outcomes (Navarro‐Vicente et al., 2011; Dinaux et al. 2017) so they all occur twice (a and b) in the plot. ES is the estimated risk of harm in each report and confidence interval (CI) the corresponding CI. Some reports have no harmful incidents and thus a risk of 0 and a CI of (0.0).

The forest plot in Figure 4 presents effect size of harmful incidents with 95% CI for each of the 25 samples of 22 reports. Overall risk of any harm was estimated as 0.2%. The effect sizes ranged from 0.00 to 0.81 and indicate that the variation is large. The pooled effect size for harmful incidents was inferior (0.002), and the index of heterogeneity was considerable for the reports (*I* ^2^ = 100%, *p* < .001) (Higgins et al., 2019).

### 4.4 Harm related to supine positioning

In our review, harmful incidents related to supine position with or without modifications occur (i.e. supine, lithotomy, Trendelenburg, beach chair). Nilsson (2013) noted that 6% (5/86) reported postoperative pain in the arms, 5% (4/86) pain in the heels and 3% (2/86) a bilateral pressure ulcer in stage I in the heels after supine positioning without modifications. Grant et al. (2019) noted that 75% (43/75) of those with intraoperative peripheral nerve injury were placed in a supine position without modifications.

Grant et al. (2019) also reported that 12% (9/75) of position‐related intraoperative peripheral nerve injuries occurred after lithotomy positioning, while Ulm et al. (2014) reported an occurrence of positioning‐related intraoperative peripheral nerve injury of 1% (7/831). Of these, 29% (2/7) and 14% (1/7) had lower and upper extremity intraoperative peripheral nerve injury, respectively. Furthermore, 43% (3/7) sustained minor head contusions and 14% (1/7) had a large subcutaneous ecchymosis on the right flank. A large register‐based report (Warner et al., 1994) reported 55 (1 per 3608) patients who had lower‐extremity intraoperative peripheral nerve injury persisting for more than 3 months after lithotomy positioning. The peroneal nerve was involved in 78% (43/55), sciatic nerve in 15% (8/55) and femoral nerve in 8% (4/55). While more than 50% of patients with peroneal and femoral intraoperative peripheral nerve injury regained motor function within 1 year, none of the patients with sciatic intraoperative peripheral nerve injury (8/55) regained complete motor function within that time. The same research group (Warner et al., 2000) reported that 2% (15/991) developed lower‐extremity intraoperative peripheral nerve injury within the first 7 postoperative days. The obturator nerve was affected in 5/15 patients, the lateral femoral cutaneous nerve in 4/15 and the peroneal and sciatic nerves in 3/15. All 15 patients with neuropathies had symptoms within 4 h of discontinuation of anaesthetics, 9/15 had paraesthesia, and 4/15 had dysesthesia within the sensory distribution of the affected nerve. The 4/15 and 2/15 who had paraesthesia but not pain spontaneously reported sensory disturbance. Objective weakness was not reported in any of the patients. Dinaux et al. (2017) reported that 22% of those who underwent the Miles procedure in lithotomy positioning had perineal wound infection and 14% perineal wound dehiscence up to 3 months after surgery.

Wen et al. (2014) reported that fewer than 1% (*n* = 698) had one or more positioning‐related injuries after Trendelenburg positioning. Eye injuries constituted 51% of total harm incidents, including blindness in one eye, visual disturbances and corneal foreign bodies. The remaining 49% experienced harm as intraoperative peripheral nerve injury, rhabdomyolysis and compartment syndrome. Grant et al. (2019) noted that 7% (5/75) had intraoperative peripheral nerve injury after Trendelenburg position. Johansson and von Vogelsang (2019) reported that 1/12 and 2/12 had pressure ulcer stage I and II, respectively, in the upper extremity, and that 1/12 had pressure ulcer stage II in the lower extremity. Furthermore, 47% (44/94) had positioning‐related symptoms of intraoperative peripheral nerve injury in the upper or lower extremities within 7–10 days after surgery. Symptoms reported in the upper extremities were numbness (8/12), tingling (8/12) and weakness (3/12), where symptoms reported in the lower extremities were numbness (5/32), tingling (2/32), weakness (12/32), sense of weight (1/32) and sensitivity of loss (6/32) (Johansson & von Vogelsang, 2019). Gezginci et al. (2015) reported symptoms of intraoperative peripheral nerve injury such as loss of sensation in the feet (3%), legs (2%), knees (1%), hands (1%) and ribs (1%). Positioning‐related postoperative pain in the back and neck (4%), upper and lower legs (3%), knees (1%), feet (1%), groin (1%), ribs (1%) and hand/arm/elbow (1%) after Trendelenburg positioning was reported. Johansson and von Vogelsang (2019) reported that 11% (9/86) had positioning‐related pain in the upper and 17% (15/86) in the lower extremity 7–10 days after Trendelenburg. Among these, 8/9 had shoulder pain, 1/9 arm pain, 9/15 pain in the legs and feet, 5/15 in the hip/pelvis and 3/15 in the thigh. Mattei et al. (2013) reported that 10%–14% had muscle pain in the upper and lower limbs after Trendelenburg positioning.

Ten patients met the criteria of rhabdomyolysis with serum creatinine kinase levels >5000 IU/L. The incidence of rhabdomyolysis ranged between 10% (7/73) and 17% (10/60) on Day 1 after Trendelenburg positioning, although none of these patients developed renal failure (Mattei et al., 2013).

Levy et al. (2019) reported that 2% (6/400) of patients had intraoperative peripheral nerve injury of the lateral femoral cutaneous nerve after beach chair positioning, where symptoms resolved within 6 months.

### 4.5 Harm related to prone positioning

Studies with patients placed in prone positioning (8 study samples) had the highest risks of harm varying from 0.19 to 0.81, with an overall risk of 0.33. In two reports (Jeon et al., 2007; Mohammadi & Hosseini, 2008), patients underwent lumbar spine surgery in the prone position and the head was placed neutral or down and with the neck in midline positioning without external direct compression to the eyes. In patients with head‐down 59% and 68% had chemosis in the two studies compared to 35% and 41% in those with neutral head positioning, respectively. A meta‐analysis gave overall RR = 1.64; 95% CI = (1.25, 2.14), *p* < .001, among the patients placed in head‐neutral positioning, 46%–83% had mild chemosis, 18%–20% moderate and 4%–5% severe. Among the patients placed in head‐down positioning, 18%–31% had mild chemosis, 31%–36% moderate, 7%–14% severe. All patients had normal vision after surgery.

In three later reports, (Choi et al., 2022; Lin et al., 2017; Techanivate et al., 2020) the prevalence of perioperative pressure ulcer ranged from 23% (48/209) to 27% (82/300) in patients undergoing spine surgery in the prone position. The ulcers were localized to the face, abdomen, pelvis and extremities and the severity of the ulcers ranged from stage I to stage III. Dinaux et al. (2017) reported that 3% and 7% had perineal wound infections and perineal dehiscence, respectively, after the Miles procedure in the prone position. Grant et al. (2019) reported that 9% (7/75) had intraoperative peripheral nerve injury after prone position.

### 4.6 Harm related to lateral positioning

Grant et al. (2019) reported that 12% (9/75) of positioning‐related intraoperative peripheral nerve injuries were due to lateral positioning, whereas Ueno et al. (2020) reported that 3% developed pressure ulcers after hip surgery in lateral positioning stabilized with pelvic positioners. Yoshimura et al. (2016) reported pressure ulcers in 11% (30/277) at 24 h after surgery in those placed in park bench positioning. Of these, 97% (29/30) had pressure ulcer stage I and 3% (1/30) had pressure ulcer stage II. The locations were the lateral thorax (12/30), iliac crest (6/30), greater trochanter (5/30), lateral thorax and/or greater trochanter (4/30), lateral thorax and/or iliac crest (1/30) and other sites (2/30).

## 5 DISCUSSION

This systematic review and meta‐analysis found that harmful incidents (i.e. peripheral nerve injury, skin injury, eye injury, musculoskeletal and eye injury) after patient positioning on the operating table do occur, but the occurrence is still unclear.

### 5.1 Intraoperative peripheral nerve injury

The review indicated that intraoperative peripheral nerve injury does occur after lateral positioning and brachial plexus injury is common (Schwarzman et al., 2020). Intraoperative peripheral nerve injury might occur due to mechanical factors as stretch, crush and transection, due to modifications of patient positions (Laughlin et al., 2020).

This review showed that intraoperative peripheral nerve injury in the upper extremities was most common after Trendelenburg positioning, while in the lower extremities the injury was most common after lithotomy positioning. These positions are variations of the supine positioning, whereby the patient lies face up with the arms often tucked and padded parallel to the body. In lithotomy position, patient's legs are placed in stirrups and knees flexed with the leg on the operating table and can be graded in four levels (low, standard, high and exaggerated). In Trendelenburg positioning, the head and body are tilted 25–45° downward with the head in lowest position (Das et al., 2019; Fawcett, 2019).

This review indicated that intraoperative peripheral nerve injury in the upper extremities is related to brachial plexus, medial, radial and ulnar nerves. Brachial plexus injury is reported in Trendelenburg positioning (Bjøro et al., 2020) and is typically in the form of painless motor dysfunction (Shveiky et al. 2010; Winfree & Kline, 2005). When patients are tilted downward, they may slide down the operating table. To prevent this, shoulder braces are commonly applied which may increase brachial plexus injuries due to compression or stretch after misplace shoulder support (Das et al., 2019; Shveiky et al. 2010). This review indicated that intraoperative peripheral nerve injury in the lower extremities is related to the obturator, femoral, lateral femoral cutaneous, popliteal, peroneal and sciatic nerves. These injuries are reported on lithotomy positioning (Bjøro et al., 2020). Hyperflexion or abduction of the hip in lithotomy positioning stretch or compress the obturator and lateral femoral cutaneous nerves. While flexion of the hip, particularly with knee flexion puts the sciatic nerve on stretch (Barnett et al., 2007; Winfree & Kline, 2005). Femoral nerve injury is demonstrated by a loss of sensation over the anterior thigh and medial lower leg. When having more severe injury, weakness of the quadriceps muscle might be more pronounced, making it more difficult to stand from a seated positioning or leading to falls during attempts at ambulation (Barnett et al., 2007; Winfree & Kline, 2005). Obturator nerve injury leads to a loss of sensation of the thigh and some experience of pain within the same sensory distribution thigh. Rare but severe harm may cause motor weakness of the adductor muscles of the thigh (Barnett et al., 2007), but this was not reported in our review. Damage to the sciatic nerve may involve peroneal division and appear as a loss of cutaneous sensation in the lateral and anterior parts of the lower leg. Harm that is more serious may result in instability to the dorsiflexion of the foot with ‘footdrop’ (Barnett et al., 2007; Winfree & Kline, 2005). Our review only reported sensory symptoms of sciatic and peroneal nerve injury, indicating less severe harm (Martial et al., 2019). Reports have reported symptoms related to upper and lower‐extremity nerve injury that resolved within 9–12 months. That indicates injury severe enough to result in demyelination and axon degeneration distal to the level of injury (Wallerian degeneration) showed that complete recovery from such harm may take up to 1 year (Martial et al., 2019).

### 5.2 Skin injury

Perioperative pressure ulcer was noted in eight reports. The updated National Pressure Ulcer Advisory Panel define pressure ulcer as localized damage to the skin and/or underlying soft tissues, usually over a bony prominence or related to a medical or other device (Edsberg et al., 2016). In this review, the stages of the ulcers were graded according to the updated National Pressure Ulcer Advisory Panel system (Edsberg et al., 2016). The reports described mainly pressure ulcer stage I (2%–10%) and stage II (3%–10%). Pressure ulcer stage I includes intact skin with non‐blanchable redness, while stage II includes partial thickness loss of the dermis presenting as a shallow open ulcer with a red/pink wound bed without slough (Edsberg et al., 2016). Hence, neither stage I nor stage II are deep ulcers. However, 71% (5/7) of those in lateral positioning with pelvic stabilization and 2% (2/82) of those in prone positioning reported perioperative pressure ulcer stage III, including full‐thickness tissue loss with visible subcutaneous fat (Edsberg et al., 2016).

An interesting finding was that incidence of perineal wound infection and wound dehiscence were higher in those in lithotomy than in prone positioning. This may be due to the better visualization of the operative field when a patient is in the prone position. Another cause may be the difficulty in maintaining sterility in lithotomy positioning (Fawcett, 2019).

### 5.3 Eye injury

Eye injuries after prone and Trendelenburg positioning were chemosis, blindness, visual disturbance and corneal foreign bodies, but such injuries are rare. Several surgical procedures (e.g. robotic‐assisted laparoscopic prostatectomy, colorectal and gynaecological robotic‐assisted laparoscopic surgery) require Trendelenburg position with the head tilted downwards 25–45°. The position uses gravity to pull the abdominal viscera from the operative field. This position might have negative physiological effects with intraocular pressure and compression of the vasculature, resulting in retinal ischemia and vision loss (Ripa et al., 2022). The meta‐analysis showed that patients placed in prone position with head‐down had higher risk of chemosis compared those with neutral head positioning. A possible explanation for this may be direct or indirect pressure on the eye, which increases intraocular pressure with preorbital swelling, vision loss or blindness (Stambough et al., 2007).

### 5.4 Musculoskeletal and vascular injury

The review showed that patients reported pain in the arms, shoulders, back, neck, pelvic, limbs and shoulder up to 10 days postoperatively. Myalgia is common after surgery (89%), and one reason for this may be patient positioning during surgery. The duration varies, usually lasting 2–3 days, but sometimes even 1 week (Gefen et al., 2020). Less severe harm, such as head contusions and subcutaneous ecchymosis, and more serious harm, such as compartment syndrome, were reported after Trendelenburg positioning with lithotomy. Compartment syndrome is a condition which increases internal pressure within the upper or lower leg compartments resulting in insufficient blood supply to tissue with muscle and nerve impairment (Satos et al., 2021). Compartment syndrome might follow long‐lasting operations in high‐lithotomy position and may occur even with careful positioning and attention to areas under pressure (Satos et al., 2021). Compartment syndrome may progress to rhabdomyolysis (Gawronski, 2019). Rhabdomyolysis was reported in 10%–17% of injuries after Trendelenburg positioning with lithotomy. Rhabdomyolysis is an injury in the sarcolemma of the muscle that results in leakage of its components into the blood or urine and is confirmed by increase in serum creatinine kinase >1000 U/L (Ahmad et al., 2021; Gawronski, 2019). A potentially life‐threatening harm of acute rhabdomyolysis is hyperkalaemia with cardiac conduction disturbance, arrhythmias, or skeletal muscle paralysis. Renal failure may develop because of intraocular obstruction and acute tubular necrosis (Ahmad et al., 2021; Gawronski, 2019). This was not reported in our review.

### 5.5 Limitations

The review has some limitations. Extraction and systematization of data from different papers are challenging and lead to some limitations in all reviews. The lack of definitions of harms and a precise description of patient positioning were often not standardized in the reports. This may suggest hidden figures of harmful incidents. There is a need to establish agreed‐upon definitions of harms, as well as how to report these, to obtain a more precise picture of injuries as a basis to improve patient safety in the field of patient positioning on the operating table. Recording harmful incidents depends on injuries, pain and other symptoms being reported and disability being recorded by standardized prospective tools for reporting harm; this was not used in the reports included in this review. The moderate to critical certainty of the evidence in this review represents limitations. Therefore, there is a need for high‐quality, well‐designed studies, to improve our knowledge of harmful incidents related to patient positioning on operating table.

## 6 CONCLUSION

This systematic review and meta‐analysis reported that harmful incidents related to patient positioning on the operating table are most common in prone position. The occurrence is still unclear, but nevertheless harm occurs in terms of intraoperative peripheral nerve injury, musculoskeletal and vascular, eye and skin injuries. The consequences of harm can be severe and lead to suffering in terms of pain and other symptoms, as well as impairment and disability. Harmful incidents related to patient positioning have been poorly investigated. There is a need for high‐quality and well‐designed studies to investigate the occurrence of harmful incidents and how to reduce harmful incidents related to patient positioning.

## 7 RELEVANCE TO CLINICAL PRACTICE

Our review indicates that occurrence of harmful incidents related to patient positioning on operating table is rare, but do occur. However, the consequences can be severe and lead to major suffering among the patients and professionals as second victims (Nydoo et al., 2020).

During the last decades, robotic‐assisted surgery has increased exponentially. In spite of the fact that robotic‐assisted laparoscopic surgery is less stressful for the patient, the positions often are complex. In addition, during robotic‐assisted laparoscopic surgery the positioning of the patients cannot be changed once the robot is docked (Fawcett, 2019; Maerz et al., 2017). The operating room teams must be aware of extreme positions and be careful when planning thorough perioperative assessments of all patients positioned for surgery. A good routine is to position and protect exposed places on the body such as the bones, joints and peripheral nerves, as well as the skin. Furthermore, it is essential to prevent stretching the peripheral nerves beyond normally tolerated limits. Avoidance of direct compression is also important, and pressure should be distributed over an area as large as possible. Many types of padding materials (e.g. blankets, towels, foam, gels and pads) are recommended (Das et al., 2019; Gefen et al., 2020). Operating room teams should also pay attention to the length of time patients have been in lithotomy positioning. After 2–3 h, the patient should be placed in supine positioning to relieve and rest the extremities in lithotomy positioning (Bjøro et al., 2022). When steep Trendelenburg positioning is used, the operating room teams reverse the tilt of the operating room table when not required by the surgical technique which decreases intraocular pressure (Ripa et al., 2022). The standard of operating room care requires ‘head‐to‐toe, front‐to‐back’ assessment. This should be conducted both pre‐ and postoperatively to identify harm. It would also be beneficial if the operating room teams were able to inform the patients preoperatively and interview them postoperatively to record whether they have experienced any harm (Bjøro et al., 2022).

To handle risk in the operating room settings there is a need to manage unexpected situations and avoid simplifications (Weick & Sutcliffe, 2015). Hence, it is important that team members do not consider positioning harm to be ‘acceptable’ or ‘normal’ in terms of viewing it as an expected part of the surgery or as a complication. Such explanations may hide the existence of larger problems (Weick & Sutcliffe, 2015). There is evidence that subordinates are more likely to report good than bad news. Operating room teams should be vigilant and report close calls and incidents as early as possible so that they can learn more and help to prevent incidents before they develop (Weick & Sutcliffe, 2015).

## AUTHOR CONTRIBUTIONS

**Signe Berit Bentsen:** Conceptualization, methods, investigation, data curation, writing – original draft, review and editing. **Geir Egil Eide:** Methods, writing – review and editing. **Siri Wiig:** Conceptualization, writing – review and editing. **Tone Rustøen:** Methods, writing – review and editing. **Cathrine Heen:** Conceptualization, writing – review and editing. **Benedikte Bjøro:** Conceptualization, methods, investigation, data curation, writing – review and editing. **Signe Berit Bentsen, Siri Wiig, Tone Rustøen, Geir Egil Eide, Cathrine Heen, Benedikte Bjøro:** Agreed to be accountable for all aspects of the work in ensuring that questions related to the accuracy or integrity of any part of the work are appropriately investigated and resolved.

## FUNDING INFORMATION

This research received no specific grant from any funding agency in public, commercial or non‐profit sectors.

## CONFLICT OF INTEREST STATEMENT

The authors declare that they have no conflicts of interest.

## Supporting information


Appendix S1.

Appendix S2.

## Data Availability

Data sharing not applicable to this article as no datasets were generated or analysed during the current study.

## References

[jan16049-bib-0001] Ahmad, S. , Anees, M. , Elahi, I. , & Mateen, F. (2021). Rhabdomyolysis leading to acute kidney injury. Journal of the College of Physicians and Surgeons–Pakistan, 31(2), 235–237. 10.29271/jcpsp.2021.02.235 33645199

[jan16049-bib-0002] Barnett, C. , Hurd, W. , Rogers, R. , Williams, N. , & Shapiro, A. (2007). Laparoscopic positioning and nerve injuries. Journal of Minimally Invasive Gynecology, 14, 664–672. 10.1016/jmig.2007.04.08 17848335

[jan16049-bib-0003] Bates, D. , Levine, D. , Salmasian, H. , Syrowatka, S. , Shahian, D. , Lipsitz, S. , Zebrowski, J. , Myers, S. , Logan, M. , Roy, C. , Iannaccone, C. , Frits, M. , Volk, L. , Dulgarian, S. , Amato, M. , Edrees, H. , Sato, L. , Folcarelli, P. , Einbinder, J. , … Mort, E. (2023). The safety of inpatient health care. The New England Journal of Medicine, 388(2), 142–153. 10.1056/NEJMsa2206117 36630622

[jan16049-bib-0004] Bjøro, B. , Ballestad, I. , Rustøen, T. , Fosmark, M. , & Bentsen, S. (2022). Positioning patients for robotic‐assisted surgery: A qualitative study of operating room nurses' experiences. Nursing Open, 10, 469–478. 10.1002/nop2.1312 36631733 PMC9834175

[jan16049-bib-0005] Bjøro, B. , Mykkeltveit, I. , Rustøen, T. , Altinbas, B. , Røyise, O. , & Bentsen, S. (2020). Intraoperative peripheral nerve injury related to lithotomy positioning with seep Trendelenburg in patients undergoing robotic‐assisted laparoscopic surgery—A systematic review. Journal of Advanced Nursing, 76, 490–503. 10.1111/jan.14271 31736124

[jan16049-bib-0006] Bohrer, J. , Walters, M. , Park, A. , Polston, D. , & Barber, M. (2009). Pelvic nerve injury following gynecologic surgery: A prospective cohort study. American Journal of Obstetrics and Gynecology, 201(531), e1–e7. 10.1016/j.ajog.2009.07.023 19761997

[jan16049-bib-0007] Brooker, K. , Vikan, M. , & Thyrli, B. (2020). A qualitative exploratory study of Norwegian OR nurses' patient positioning priorities. AORN Journal, 111(2), 2011–2020. 10.1002/aorn.12930 31997315

[jan16049-bib-0008] Choi, S. , Kim, Y. , Oh, H. , Lee, C. , Yang, S. , Kim, C. , Chung, C. , & Park, H. (2022). Factors associated with perioperative hospital acquired pressure injury in patients undergoing spine surgery in the prone position: A prospective observational study. Journal of Neurosurgical Anesthesiology, 36(1), 45–52. 10.1097/ANA.0000000000000867 36006663

[jan16049-bib-0009] Cornelius, J. , Mudlagk, J. , Afferi, L. , Baumeister, P. , Mattei, A. , Moschini, M. , Iselin, C. , & Mordasini, L. (2021). Postoperative peripheral neuropathies associated with patient positioning during robot‐assisted laparoscopic radical prostatectomy (RARP): A systematic review. The Prostata, 81, 361–367. 10.1002/pros.24121 33764601

[jan16049-bib-0010] Das, D. , Propst, K. , Wechter, M. , & Kho, R. (2019). Evaluation of positioning devices for optimalization of outcomes in laparoscopic and robotic‐assisted gynecologic surgery. Journal of Minimally Invasive Gynecology, 26(2), 244–252. 10.1016/j.jmig.2018.08.027 30176363

[jan16049-bib-0011] Dinaux, A. , Amri, R. , & Berger, D. (2017). Prone positioning reduces perineal infections when performing the miles procedure. American Journal of Surgery, 214, 217–221. 10.1016/j.amjsurg.2017.05.021 28610935

[jan16049-bib-0012] Edsberg, L. , Black, J. , Goldberg, M. , McNichol, L. , & Seiggren, M. (2016). Revised national pressure ulcer advisory panel pressure injury staging system: Revised pressure injury staging system. Journal of Wound, Ostomy, and Continence Nursing, 43(6), 585–597. 10.1097/won.0000000000000281 PMC5098472 27749790

[jan16049-bib-0013] Fawcett, D. (2019). Positioning the patient for surgery. In I. J. Rothrock & D. McEwen (Eds.), Alexander's care of the patient in surgery (16th ed., pp. 142–175). Elsevier.

[jan16049-bib-0014] Gawronski, D. (2019). Trauma surgery. In J. Rothorock & D. McEwen (Eds.), Alexander's care of the patient in surgery (16th ed., pp. 1092–1118). Elsevier.

[jan16049-bib-0015] Gefen, A. , Creehan, S. , & Black, J. (2020). Critical biomechanical and clinical insights concerning tissue protection when positioning patients in the operating room: A scoping review. International Wound Journal, 17, 1405–1423. 10.1111/iwj.13408 32496025 PMC7948884

[jan16049-bib-0016] Gezginci, E. , Ozkaptan, O. , Yalcin, S. , Akin, Y. , Rassweiler, J. , & Gozen, A. (2015). Postoperative pain and neuromuscular complications associated with patient positioning after robotic assisted laparoscopic radical prostatectomy: A retrospective non‐placebo and non‐randomized study. International Urology and Nephrology, 47, 1635–1641. 10.1007/s11255-015-1088-8 26329741

[jan16049-bib-0017] Grant, I. , Browman, E. , Kang, D. , Greenberg, P. , Saba, R. , & Urman, R. (2019). A medicolegal analysis of positioning‐related perioperative peripheral nerve injuries occurring between 1996 and 2015. Journal of Clinical Anesthesia, 58, 84–90. 10.1016/j.jclinane.2019.05.013 31128482

[jan16049-bib-0018] Higgins, J. , Thomas, J. , Chandler, J. , Cumpston, M. , Li, T. , Page, M. , & Welch, V. (2019). Cochrane handbook for systematic reviews of interventions (2nd ed.). Wiley Blackwell.

[jan16049-bib-0019] Jeon, Y. , Park, Y. , Wonhwang, J. , Lim, Y. , Oh, Y. , & Park, H. (2007). Effect of head position on postoperative chemosis after prone spinal surgery. Journal of Neurosurgical Anesthesiology, 19(1), 1–4. 10.1097/01.ana.0000211024.41797.b5 17198093

[jan16049-bib-0020] Jha, A. , Prasopa‐Plaizer, N. , Larizgoita, I. , & Bates, B. (2010). Patient safety research: An overview of the global evidence. Quality & Safety in Health Care, 19, 42–47. 10.1136/qshc.2008.029165 20172882

[jan16049-bib-0021] Johansson, V. , & von Vogelsang, A. (2019). Patient‐reported extremity symptoms after robot‐assisted laparoscopic cystectomy. Journal of Clinical Nursing, 28, 1708–1718. 10.1111/jocn.14781 30653776

[jan16049-bib-0023] Laughlin, R. , Johnson, R. , Burkle, C. , & Staff, N . (2020). Postsurgical neuropathy: A descriptive review. Mayo Clinic Proceedings, 95(2), 355–369. 10.1016/j.mayocp.2019.05.038 32029088

[jan16049-bib-0024] Levy, J. , Tauberg, B. , Holtzman, A. , & Gruson, K. (2019). Reducing lateral femoral cutaneous nerve palsy in obese patients in the beach chair position: Effect of a standardized positioning and padding protocol. The Journal of the American Academy of Orthopaedic Surgeons, 27, 437–443. 10.5435/JAAOS-D-17-00624 30325879

[jan16049-bib-0025] Lin, S. , Hey, H. , Lau, E. , Tan, K. , Lau, L. , Kumar, N. , & Wong, H. (2017). Prevalence and predictors of pressure injuries from spine surgery in the prone position. Spine, 42(22), 1730–1736. 10.1097/BRS.0000000000002177 28368987

[jan16049-bib-0026] Maerz, D. , Beck, L. , Sim, A. , & Gainsburg, D. (2017). Complications of robotic‐assisted laparoscopic surgery distant from the surgical site. British Journal of Anaesthesia, 118(4), 492–503. 10.1093/bja/aex003 28403397

[jan16049-bib-0027] Martial, C. , Laurence, R. , Jean‐Michel, J. , Alexis, V. , & Fabrice, B. (2019). Peripheral nerve regeneration and intraneural revascularization. Neural Regeneration Research, 14(1), 24–33. 10.4103/1673-5374.243699 30531065 PMC6263011

[jan16049-bib-0028] Mattei, A. , DiPierro, G. , Rafeld, B. , Konrad, C. , Beutler, J. , & Danuser, H. (2013). Positioning injury, rhabdomyolysis, and serum creatine kinase‐concentration course in patients undergoing robot‐assisted radical prostatectomy and extended pelvic lymph node dissection. Journal of Endourology, 27(1), 45–51. 10.1089/end.2012.0169 22770120

[jan16049-bib-0029] Mills, J. , Burris, M. , Warburton, D. , Conaway, M. , Schenkman, N. , & Krupski, T. (2013). Positioning injuries associated with robotic assisted urological surgery. The Journal of Urology, 190, 580–584. 10.1016/j.juro.2013.02.3185 23466240

[jan16049-bib-0030] Mohammadi, S. , & Hosseini, A. (2008). Effects of head position on postoperative conjunctival swelling after prone spinal surgery. Journal of Medical Sciences, 8(1), 44–48. 10.3923/jms.2008.44.48

[jan16049-bib-0031] Navarro‐Vicente, F. , Garicia‐Granero, A. , Frasson, M. , Blanco, F. , Flor‐Lorente, B. , Garica‐Botello, S. , & Garcia‐Granero, E. (2011). Prospective evaluation of intraoperative peripheral nerve injury in colorectal surgery. Colorectal Disease, 14, 382–385. 10.1111/j.1463-1318.2011.02630.x 21689319

[jan16049-bib-0032] Nilsson, U. G. (2013). Intraoperative positioning of patients under general anesthesia and the risk of postoperative pain and pressure ulcers. Journal of Perianesthesia Nursing, 28(3), 137–143. 10.1016/j.jopan.2012.09.006 23711309

[jan16049-bib-0033] Nydoo, P. , Pillay, B. , Naicker, T. , & Moodley, J. (2020). The second victim phenomenon in health care: A literature review. Scandinavian Journal of Public Health, 48(6), 629–637. 10.1177/1403494819855506 31405351

[jan16049-bib-0034] Oblak, T. , & Gillespie, B. (2021). The incidence of peripheral nerve injuries related to patient positioning during robotic assisted surgery: An evidence summary. Journal of Perioperative Nursing, 34(1), e49–e53. 10.26550/2209-1092.1166

[jan16049-bib-0035] Page, M. , McKenzie, J. , Bossuyt, P. , Boutron, I. , Hoffmann, T. , Mulrow, C. , Shamseer, L. , Tetzlaff, J. , Akl, E. , Brennan, S. , Chou, R. , Glanville, J. , Grimshaw, J. , Hróbjartsson, A. , Lalu, M. , Li, T. , Loder, E. , Mayo‐Wilson, E. , McDonald, S. , … Moher, D. (2021). The PRISMA 2020 statement: An updated guideline for reporting systematic reviews. BMJ Open, 372, n71. 10.1136/bmj.n71 PMC8005924 33782057

[jan16049-bib-0036] Ripa, M. , Schipa, C. , Kopsacheilis, N. , Nomikarios, M. , Perrota, G. , DeRosa, C. , Aceto, P. , Sollazzi, L. , DeRosa, P. , & Motta, L. (2022). The impact of steep Trendelenburg position on intraocular pressure. Journal of Clinical Medicine, 11, 2844–2855. 10.3390/jcm1102844 35628970 PMC9146028

[jan16049-bib-0037] Satos, H. , Kotani, Y. , Takamatsu, S. , Ohta, M. , Shiro, R. , Yamamoto, K. , Murakami, K. , & Matsumura, N. (2021). Lower leg compartment syndrome following laparoscopic uterine malignancy surgery for uterine cancer complicated by rheumatoid arthritis: A case report and literature review. European Journal of Gynaecological Oncology, 42(3), 590–594. 10.31083/j.ejgo.2021.03.2296

[jan16049-bib-0038] Schwarzman, G. , Schwarzman, L. , Macgillis, K. , & Gonzalez, M. (2020). A systematic review of upper extremity neuropathy following total hip arthroplasty. Hip International, 30(6), 673–678. 10.1177/1120700019901268 31971022

[jan16049-bib-0039] Shveiky, D. , Aseff, J. N. , & Iglesia, C. B. (2010). Brachial plexus injury after laparoscopic and robotic surgery [review]. Journal of Minimally Invasive Gynecology, 17(4), 414–420. 10.1016/j.jmig.2010.02.010 20621005

[jan16049-bib-0040] Stambough, J. , Dolan, D. , Werner, R. , & Godfrey, E. (2007). Ophthalmologic complications associated with prone positioning in spine surgery. The Journal of the American Academy of Orthopaedic Surgeons, 15, 156–165. 10.5435/00124635-200703000-00005 17341672

[jan16049-bib-0041] Sterne, J. , Hernán, M. , Reeves, B. , Savović, J. , Berkman, N. , Viswanathan, M. , Henry, D. , Altman, D. , Ansari, M. , Boutron, I. , Carpenter, J. , Chan, A. , Churchill, R. , Deeks, J. , Hróbjartsson, A. , Kirkham, J. , Jüni, P. , Loke, Y. K. , Pigott, T. , … Higgins, J. (2016). ROBINS‐I: A tool for assessing risk of bias in non‐randomised studies of interventions. BMJ, 355, i4919. 10.1136/bmj.i4919 27733354 PMC5062054

[jan16049-bib-0042] Sterne, J. , Savović, J. , Page, M. , Elbers, R. , Blencowe, N. , Boutron, I. , Cates, C. , Cheng, H. , Corbett, M. , Eldridge, S. , Emberson, J. , Hernán, M. , Hopewell, S. , Hróbjartsson, A. , Junqueira, D. , Jüni, P. , Kirkham, J. , Lasserson, T. , Li, T. , … Higgins, J. (2019). RoB 2: A revised tool for assessing risk of bias in randomised trials. BMJ, 366, l4898. 10.1136/bmj.4898 31462531

[jan16049-bib-0043] Techanivate, A. , Athibai, N. , Siripongsaporn, S. , & Singhatanadigige, W. (2020). Risk factors for facia pressure ulcers in patients who underwent prolonged prone orthopedic spine surgery. Spine, 46(11), 744–750. 10.1097/BRS.0000000000003892 33337680

[jan16049-bib-0044] Tsubouchi, K. , Gunge, N. , Tominaga, K. , Matsuzaki, H. , Fujikawa, A. , Emoto, T. , Miyazaki, T. , Okabe, Y. , Nakamura, N. , Kataoka, M. , Ogawa, S. , Akaihata, H. , Sato, Y. , Hata, J. , Matsuoka, H. , Kojima, Y. , & Haga, N. (2022). Efficacy of the opened legs position for protecting against postoperative rhabdomyolysis after robot‐assisted radical prostatectomy: A propensity score‐matched analysis of perioperative outcomes. International Journal of Urology: Official Journal of the Japanese Urological Association, 29, 1132–1138. 10.1111/iju.14935 35606052

[jan16049-bib-0045] Ueno, T. , Kabata, T. , Kajino, Y. , Inoue, D. , Ohmori, T. , Yoshitani, J. , Ueoka, K. , Yamamuro, Y. , & Tsuchiya, H. (2020). Risk factors for pressure ulcers from the use of a pelvic positioner in hip surgery: A retrospective observational cohort study in 229 patients. BMC Anesthesiology, 14(10), 1–10. 10.1186/s13037-020-00237-7 PMC7137331 32280374

[jan16049-bib-0046] Ulm, M. , Fleming, N. , Rallapali, V. , Munsell, M. , Ramirez, P. , Westin, S. , Nick, A. , Schmeler, K. , & Soliman, P. (2014). Position‐related injury is uncommon in robotic gynecologic surgery. Gynecologic Oncology, 135, 534–538. 10.1016/j.ygyno.2014.10.016 25449565 PMC4268144

[jan16049-bib-0047] Warner, M. , Martin, J. , Schroeder, D. , Offord, K. , & Chute, C. (1994). Lower‐extremity motor neuropathy associated with surgery performed on patients in a lithotomy position. Anesthesiology, 81, 6–12. 10.1097/00000542-199407000-00004 8042811

[jan16049-bib-0048] Warner, M. , Warner, D. , Harper, M. , & Schroeder, D. (2000). Lower extremity neuropathies associated with lithotomy positions. Anesthesiology, 93, 938–942. 10.1097/00000542-200010000-00010 11020742

[jan16049-bib-0049] Weick, K. , & Sutcliffe, K. (2015). Preoccupation with failure. In K. Weick & K. Sutcliffe (Eds.), Managing the unexpected. Sustained performance in a complex world (3rd ed., pp. 45–61). Wiley.

[jan16049-bib-0050] Wen, T. , Deibert, C. , Siringo, F. , & Spencer, B. (2014). Positioning‐related complications of minimally invasive radical prostatectomies. Journal of Endourology, 28(6), 660–667. 10.1089/end.2013.0623 24428586

[jan16049-bib-0051] Winfree, C. J. , & Kline, D. G. (2005). Intraoperative positioning nerve injuries [review]. Surgical Neurology, 63(1), 5–18. http://ovidsp.ovid.com/ovidweb.cgi?T=JS&CSC=Y&NEWS=N&PAGE=fulltext&D=med5&AN=15639509 15639509 10.1016/j.surneu.2004.03.024

[jan16049-bib-0052] World Health Organization (WHO) . (2005). World alliance for patient safety. WHO draft guidelines for adverse event reporting and learning systems. From information to action. Retrieved September 13, 2022, from https://apps.who.int/iris/bitstream/handle/10665/69797/WHO‐EIP‐SPO‐QPS‐05.3‐eng.pdf

[jan16049-bib-0053] World Health Organization (WHO) . (2019). Patient safety. Retrieved September 13, 2022, from https://www.who.int/news‐room/fact‐sheets/detail/patient‐safety

[jan16049-bib-0054] Yoshimura, M. , Lizaka, S. , Kohno, M. , Nagata, O. , Yamasaki, T. , Mae, T. , Haruyama, N. , & Sanada, H. (2016). Risk factors associated with intraoperatively acquired pressure ulcers in the park‐bench position: A retrospective study. International Wound Journal, 13, 1206–1213. 10.1111/iwj.12445 26043765 PMC7949538

