# Supplementary material for: Patient positioning on the operating table and patient safety: A systematic review and meta‐analysis
Source: J Adv Nurs. 2024 Jan 7;81(9):5585–602. doi: 10.1111/jan.16049 (PMC12371821; doi:10.1111/jan.16049)
Supplement: Supplementary file 2 — Appendix S2. [file JAN-81-5585-s002.docx]

APPENDIX: 2

Documentation on literature search for:

Patient positioning on operating table and patient safety: A systematic review 18.08.2023.

**Medline**

**Database: Ovid MEDLINE(R) and Epub Ahead of Print, In-Process, In-Data-Review & Other Non-Indexed Citations and Daily <1946 to August 17, 2023>**
**Search Strategy:**
**1**  exp Patient Positioning/ (8597)
**2**  (patient* adj2 position*).ti,ab. (10941)
**3**  1 or 2 (18204)
**4**  ((surg* or operat*) adj (table* or room*)).ti,ab. (37976)
**5**  exp Patient Safety/ (25480)
**6**  exp Quality Improvement/ (33757)
**7**  exp "Quality of Health Care"/ (8158542)
**8**  exp Risk Management/ (347963)
**9**  exp Accident Prevention/ (95679)
**10**  exp Intraoperative Complications/ (56854)
**11**  exp Safety/ (88879)
**12**  exp Safety Management/ (21478)
**13**  exp Equipment Failure/ (91065)
**14**  exp Equipment Safety/ (10453)
**15**  exp Crew Resource Management, Healthcare/ (66)
**16**  ((medical or medicine* or patient* or clinical*) and (safety or error*)).ti,ab. (601487)
**17**  (adverse* adj event*).ti,ab. (222738)
**18**  (accident* adj2 prevention*).ti,ab. (1585)
**19**  5 or 6 or 8 or 9 or 10 or 11 or 12 or 13 or 14 or 15 or 16 or 17 or 18 (1263745)
**20**  3 and 4 and 19 (140)
**21**  limit 20 to yr="2023 -Current" (6)

**Embase**

**Database: Embase <1974 to 2023 Week 32>**
**Search Strategy:**
**1**  exp patient positioning/ (23415)
**2**  (patient* adj2 position*).ti,ab. (18390)
**3**  1 or 2 (36559)
**4**  ((surg* or operat*) adj (table* or room*)).ti,ab. (55835)
**5**  exp patient safety/ (164721)
**6**  exp total quality management/ (90548)
**7**  exp health care quality/ (4178449)
**8**  exp risk management/ (65433)
**9**  exp accident prevention/ (16941)
**10**  exp peroperative complication/ (59053)
**11**  exp safety/ (566493)
**12**  exp device failure/ (8351)
**13**  exp device safety/ (20944)
**14**  exp health care personnel management/ (3986)
**15**  ((medical or medicine* or patient* or clinical*) and (safety or error*)).ti,ab. (998250)
**16**  (adverse* adj event*).ti,ab. (394556)
**17**  (accident* adj2 prevention*).ti,ab. (1785)
**18**  5 or 6 or 7 or 8 or 9 or 10 or 11 or 12 or 13 or 14 or 15 or 16 or 17 (5345026)
**19**  3 and 4 and 18 (364)
**20**  limit 19 to yr="2023 -Current" (15)

**Cinahl**

| **#** | **Query** | **Results** |
| --- | --- | --- |
| S1 | (MH "Patient Positioning+") | 14,967 |
| S2 | TI patient* W1 position* OR AB patient* W1 position* | 2,105 |
| S3 | S1 OR S2 | 16,187 |
| S4 | TI ( (surg* or operat*) W0 (table* or room*) ) OR AB ( (surg* or operat*) W0 (table* or room*) ) | 11,960 |
| S5 | (MH "Patient Safety+") | 145,951 |
| S6 | (MH "Quality Improvement+") | 77,208 |
| S7 | (MH "Quality of Health Care+") | 922,822 |
| S8 | (MH "Risk Management+") | 21,031 |
| S9 | (MH "Treatment Errors+") | 27,583 |
| S10 | (MH "Intraoperative Complications+") | 14,366 |
| S11 | (MH "Safety+") | 213,546 |
| S12 | (MH "Equipment Failure+") | 22,915 |
| S13 | (MH "Equipment Safety") | 4,922 |
| S14 | TI ( (medical or medicine* or patient* or clinical*) and (safety or error*) ) OR AB ( (medical or medicine* or patient* or clinical*) and (safety or error*) ) | 183,006 |
| S15 | TI adverse* W0 event* OR AB adverse* W0 event* | 72,293 |
| S16 | TI accident* W2 prevention* OR AB accident* W2 prevention* | 358 |
| S17 | S5 OR S6 OR S7 OR S8 OR S9 OR S10 OR S11 OR S12 OR S13 OR S14 OR S15 OR S16 | 1,241,005 |
| S18 | S3 AND S4 AND S17 | 113 |
| S19 | S3 AND S4 AND S17 | 5 |

**Cochrane**


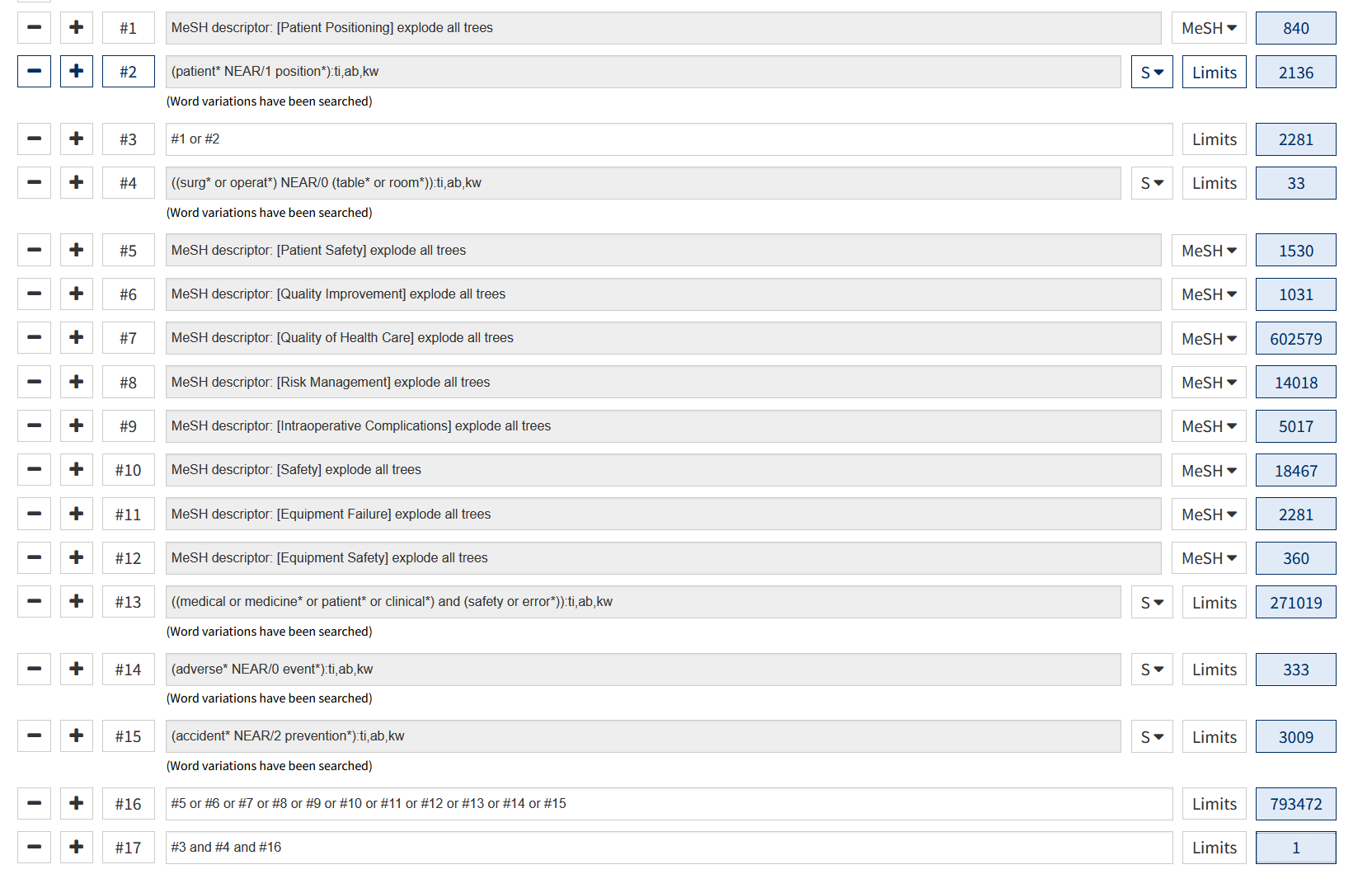


**Epistemonikos**

«patient positioning»

«positioning of patient»

«position patient»

«patient position»

**Scopus**


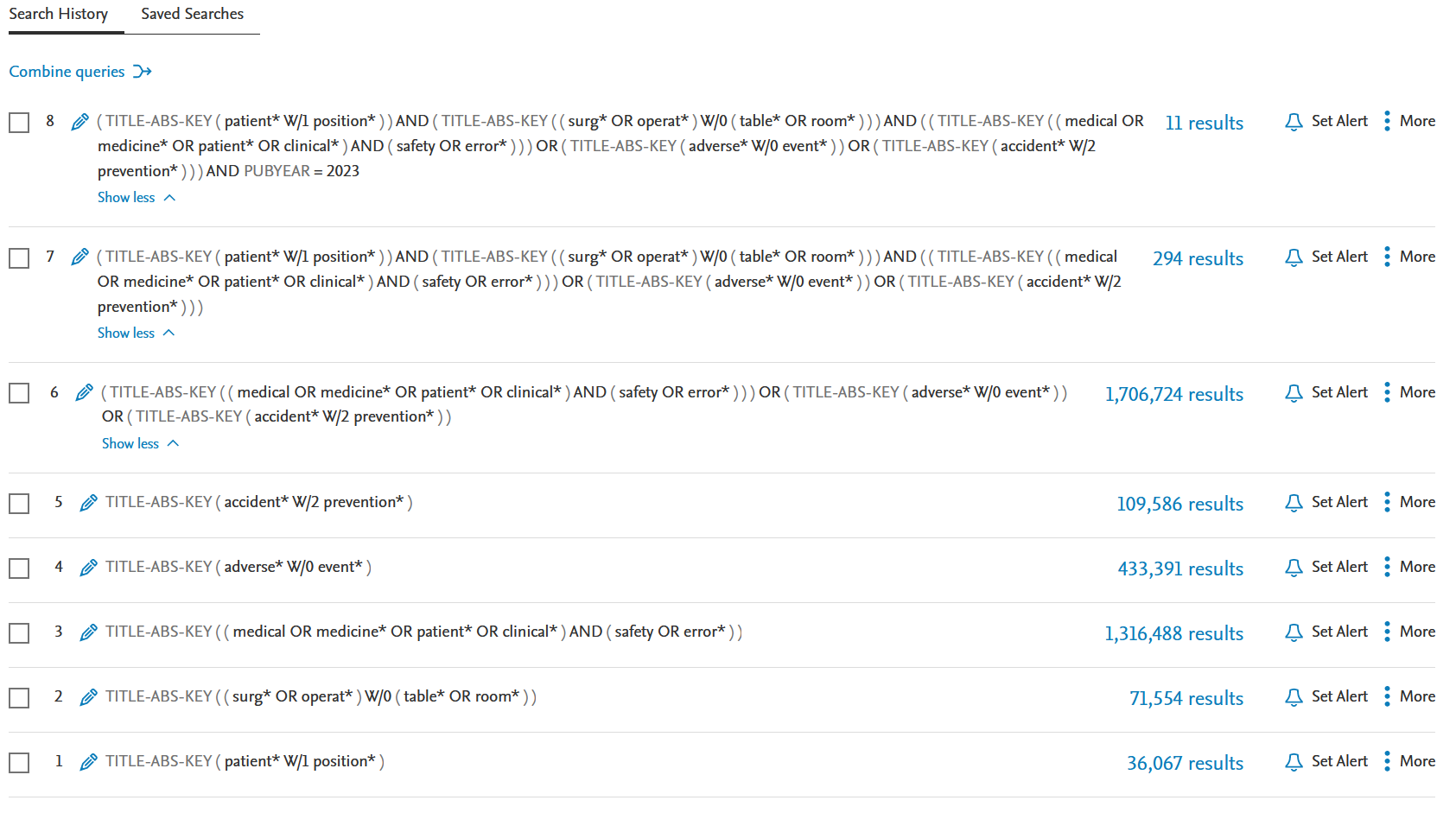


**Web of Science**


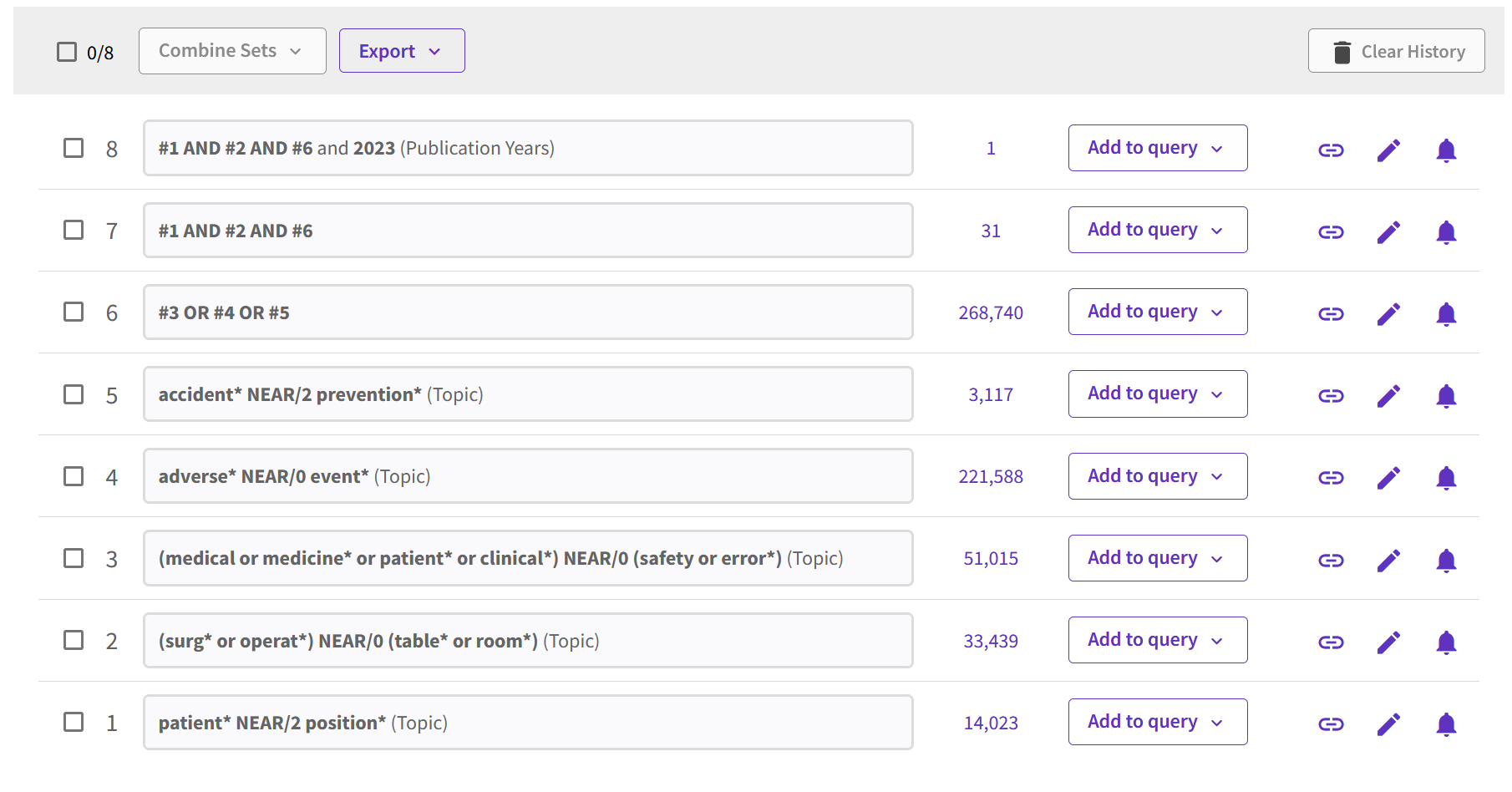


**Google Scholar**

"patient positioning" operation|surgery "patient safety"

"patient positioning" operation|surgery «medical error»

"patient positioning" operation|surgery «adverse events»

"patient positioning" operation|surgery «accident prevention»
